# Patterns of Dickkopf-3 Serum and Urine Levels at Different Stages of Chronic Kidney Disease

**DOI:** 10.3390/jcm12144705

**Published:** 2023-07-15

**Authors:** Paulina Dziamałek-Macioszczyk, Agata Winiarska, Anna Pawłowska, Paweł Wojtacha, Tomasz Stompór

**Affiliations:** 1 Department of Nephrology, Hypertension and Internal Medicine, University of Warmia and Mazury in Olsztyn, 10-561 Olsztyn, Poland; 2 Department of Industrial and Food Microbiology, University of Warmia and Mazury in Olsztyn, 10-726 Olsztyn, Poland

**Keywords:** chronic kidney disease, renal fibrosis, tubular injury, epithelial-to-mesenchymal transition

## Abstract

Dickkopf 3 (Dkk3) is a WNT/β-catenin signaling pathway regulator secreted by tubular epithelial cells upon the influence of different stressors. Recently Dkk3 was described as a biomarker of tubular cell injury and a tool that may estimate the risk of chronic kidney disease (CKD) progression. The data about Dkk3 concentrations at particular stages of CKD are lacking. The aim of this study was to measure serum and urine Dkk3 levels in patients with different ‘renal status’ and evaluate its role as a biomarker of renal damage. One hundred individuals, aged between 24 and 85 years (mean 53.1 ± 17.1), were enrolled in the study. Five groups of 20 subjects each were recruited based on their kidney function. Serum and urine Dkk3 levels were measured by ELISA. The highest median urinary Dkk3 normalized to urinary creatinine was found in patients with established CKD (7051 pg/mg). It was two times higher in renal transplant patients (5705 pg/mg) than in healthy individuals (2654 pg/mg) and the glomerulonephritis group (2470 pg/mg). Urinary Dkk3 was associated with serum creatinine in participants with established CKD and following transplantation. Our results confirm the potential role of Dkk3 as a biomarker of an ongoing renal injury.

## 1. Introduction

Fibrosis and cellular atrophy are well-recognized mechanisms leading to progressive organ failure [1,2]. If this process affects kidneys, it results in chronic kidney disease (CKD), which appears one of the most prevalent health problems worldwide and contributes to premature and excess morbidity and mortality [2,3,4]. Several proteins engaged in multiple signaling pathways are involved in CKD progression. One such pathway includes WNT/β-catenin signaling which contributes to: cellular proliferation and migration, expression of pro-fibrotic cytokines, and epithelial-to-mesenchymal transition (EMT) [3,5,6]. WNT/β-catenin pathway activation in the short term has a protective effect on tubular cells since it mitigates apoptosis and promotes tubular regeneration (for example, following acute kidney injury [AKI]) [5,7]. On the contrary, constant and prolonged activation of this pathway results in kidney fibrosis in the course of chronic nephropathies, including nephroangiosclerosis, sequelae of AKI (now classified as acute kidney disease and CKD following AKI), proteinuric kidney diseases and renal pathologies with cyst formation [5,6,7,8,9].

The role of WNT/β-catenin pathway in the pathophysiology of CKD is—among other mediators—orchestrated by members of the Dickkopf (Dkk) family proteins. One of them is Dickkopf 3 (Dkk3)—stress-induced glycoprotein secreted by renal tubular epithelial cells. Most Dkk proteins (Dkk1, Dkk2, and Dkk4) inhibit the WNT/β-catenin pathway by interaction with two receptors: lipoprotein receptor-related protein (LPR) 5 and LPR6 [5,8,10,11]. Dkk3 either activates or inhibits WNT/β-catenin signaling depending on the ‘tissue context’ [5,12,13]. In the kidney, it activates WNT/β-catenin signaling in tubular epithelial cells (with their reprogramming into proinflammatory phenotype) and promotes fibrosis via lymphocyte T activation [5,12]. A study conducted by Federico et al. revealed that Dkk3 is expressed in the developing kidney, silenced in adulthood, and then re-expressed in response to various stressors [3,12]. Other studies provided evidence that genetic and antibody-mediated deletion of the Dkk3 gene resulted in preserved kidney function in various animal models of CKD [1].

Moreover, recent studies suggest that Dkk3 may serve as the clinical biomarker of renal damage and a prognostic tool for estimating the risk of CKD progression. Schunk et al. described urinary Dkk3 as an indicator of tubular injury and predictor of both short-term glomerular filtration rate (GFR) loss as well as the long-term CKD progression, independent from albuminuria or the underlying cause of CKD in humans [1,3,5]. They also demonstrated that elevated urinary Dkk3 level is associated with an increased risk for AKI and CKD following cardiac surgery [3,5]. Few studies describing Dkk3 as a kidney outcome prognostic tool in autoimmune diseases, namelylupus, and ANCA-associated vasculitis were also published [14,15].

To the best of our knowledge no studies are available that would systematically describe the patterns of serum and urine Dkk3 concentrations in patients representing the major groups of ‘kidney’ patients, i.e., those with proteinuria in the course of biopsy-proven glomerular pathology, established CKD (with GFR permanently reduced below 60 mL/min/1.73 m^2^), kidney transplant recipients and patients with end-stage renal disease (ESRD) treated with hemodialysis. The aim of our study was to measure serum and urine Dkk3 in mentioned groups of patients and to evaluate the possible role of this protein as a biomarker of renal injury and cardiovascular risk.

## 2. Materials and Methods

### 2.1. Study Population

One hundred individuals were enrolled in our study. The mean age equaled 53.1 ± 17.1 years (range 24–85 years) and 46% were female. Five groups of subjects (20 people each) were recruited.

Group 1 (G1) of healthy individuals with normal kidney function, i.e., eGFR ≥ 60 mL/min/1.73 m^2^ and normal urine biochemistry and sediment, served as a control group. None of them was previously diagnosed with any chronic disease, including arterial hypertension (AHT) and diabetes (DM).

Group 2 (G2) consisted of patients with preserved kidney function who were admitted to our department to perform a kidney biopsy. Enrollment criteria included eGFR ≥ 60 mL/min/1.73 m^2^ and urinary albumin-to-creatinine ratio (UACR) > 0.5 g/g and/or abnormal urine sediment. All patients diagnosed with DM, a history of cardiovascular event, or prior immunosuppressive treatment were excluded (we screened 50 consecutive patients to establish the final group). Biopsy-based diagnoses were as follows: IgA nephropathy (8 patients; 40%), anti-PLA_2_R antibody-positive membranous nephropathy (5 patients; 25%), primary focal/segmental glomerulosclerosis (3 patients; 15%), type IV collagen disease (2 patients; 10%), minimal change disease and C3 nephropathy with membrano-proliferative pattern of injury (1 patient i.e., 5% each).

Group 3 (G3) comprised patients with established CKD G3-5 (for those with stage 5—not yet on dialysis). Mean time since CKD diagnosis equaled 3.5 ± 7.8 years. All patients were recruited from our outpatient department. Four patients in this group (20%) had a history of a cardiovascular event, none of them suffered from diabetes nor received immunosuppressive agents at the time of assessment. Causes of CKD were as follows: CKD attributed to cardio-renal syndrome or ischemic/hypertensive nephropathy (13 patients; 60%), chronic glomerulonephritis (3 patients; 15%), nephrolithiasis, autosomal dominant polycystic kidney disease (ADPKD), CKD following chronic pyelonephritis (1 patient or 5% each).

Individuals included in group 4 (G4) were treated in our dialysis center (mean time on dialysis 4.1 ± 7.2 years). All of them were dialyzed 3 times a week for 4.0–4.5 h using High Flux biocompatible FX-class dialyzers (Fresenius, Bad Homburg, Germany). Six patients had a history of cardiovascular events, none of them was treated with immunosuppressive drugs at the time of assessment. End-stage renal disease (ESRD) resulted from glomerulonephritis in 11 patients (55%), ischemic/hypertensive nephropathy in 4 (20%), and ADPKD in 1 (5%). In the case of 4 patients (20%) origin of ESRD remained unknown.

Group 5 (G5) comprised kidney transplant recipients (KTx) with eGFR ≥ 60 mL/min/1.73 m^2^ (mean time following KTx 3.7 ± 5.4 years and mean time on RRT before KTx 2.6 ± 1.5 years). Only one (5%) had a history of prior cardiovascular events. Immunosuppressive regimens were as follows: steroids/mycophenolate mofetil/tacrolimus (70%), steroids/mycophenolate mofetil/cyclosporine (25%), steroids/mycophenolate sodium/tacrolimus (5%).

### 2.2. Laboratory Measurements and Ardiovascular Status Assessment

Blood samples were collected from all participants. Morning (first void) urine samples were not collected only from G4 patients because of anuria or negligible urine volume. Biochemical parameters were analyzed at the certified laboratory using Cobas 6000 analyzer (Roche, Basel, Switzerland). Urine albumin used to calculate UACR was measured using immunoturbidimetric assay by the same analyzer. Blood pressure (BP) and heart rate (HR) were measured using the Omron M3 device (Kyoto, Japan) according to the ESH/ESC Guidelines. Pulse wave velocity (PWV) was measured with a Sphygmocor X-cell device (AtCor Medical Pty. Ltd., Sydney, Australia).

### 2.3. Dkk3 Measurements

Fasting blood for assessing Dkk3 and other parameters was centrifuged immediately after sampling at 4 °C with a speed of 1000× *g* for 15 to 30 min. All serum and urine samples were stored at −80 °C. Then material was thawed at −20 °C for 24 h, 6 °C for the next 24 h, and finally at room temperature for 30 min prior to mixing by a vortex shaker. Dkk3 level was assayed using Human Dickkopf 3 ELISA kit (Bioassay Technology Laboratory, Shanghai, China, Cat. No. E2065Hu). All measurements were repeated twice for each sample with a coefficient of variation (CV) of 0.84%. The CV was calculated as the ratio of the standard deviation to the mean multiplied by 100. We normalized urinary Dkk3 (uDkk3) concentrations to urinary creatinine levels to correct urine volume (uDkk3/creat.).

### 2.4. Definitions

Body mass index (BMI) was calculated as weight in kilograms divided by the square of height in meters. Arterial hypertension (AHT) was defined as a systolic blood pressure (SBP) ≥ 140 mmHg and/or diastolic blood pressure ≥ 90 mmHg and/or any anti-hypertensive therapy, according to 2019 ESH/ESC Guidelines. The estimated glomerular filtration rate (eGFR) was calculated using creatinine-based abbreviated MDRD formula. Cardiovascular event was defined as: the history of myocardial infarction and/or stroke, transient ischaemic attack and/or peripheral vascular disease, and/or diagnosis of heart failure (based on clinical data and confirmed by echocardiography and/or NT-pro-BNP). Chronic kidney disease (CKD) was diagnosed and staged according to the KDIGO criteria.

### 2.5. Statistical Analysis

All statistical analyses were performed using the SciPy library (version 1.7.1) in Python language (version 3.8.10, Python Software Foundation, Beaverton, OR, USA). Data distribution was checked with the Shapiro–Wilk test. To compare differences between 2 independent groups, Student’s *t*-test with Bonferroni correction was applied. A comparison of differences among 5 independent groups was conducted using the Kruskal–Wallis test. Spearman’s correlation coefficients were calculated to determine the association between Dkk3 and biochemical parameters. Serum and urine Dkk3 protein amounts were presented as the median and interquartile range (IQR). Values of *p* < 0.05 were considered statistically significant.

## 3. Results

The distribution of serum Dkk3 (sDkk3) values was normal only in G1, i.e., healthy individuals. In other groups it was non-normal. The highest median sDkk3 concentration was found in subjects from G1 (79 ng/mL; IQR 30–278). In G2, i.e., a proteinuric group with normal renal function, it was the lowest (34 ng/mL; IQR 30–39) and 57% lower than in G1. In order to reduce the false positive results, differences between the two groups were checked by Student’s *t*-test with Bonferroni correction. They were significant only for G2 vs. G4 (dialysis) (*p* = 0.02).

**Figure 1:**
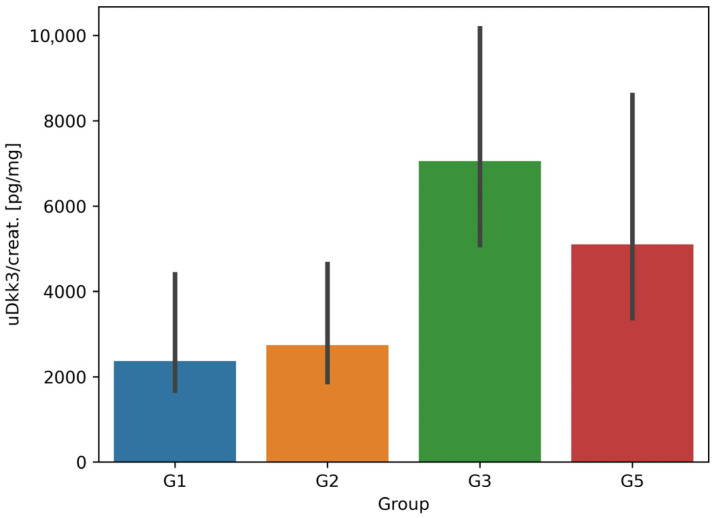
Differences between uDkk3/creat. values within study groups. Data are presented as median (boxes) and interquartile ranges (black vertical lines) because of non-normal distribution. Median uDkk3/creat. is the highest in G3. In G5 is two times higher than in G1 and G2. In G4 was not measured (urine absent or of negligible volume). For *p* values of the differences between respective groups—see text.

In case of uDkk3/creat. concentrations, data distribution was non-normal in all groups. The highest median uDkk3/creat. value was found in G3 patients (established CKD) (7050 pg/mg; IQR 5090–11,730). It was two times higher in G5 (transplant patients) (5710 pg/mg; IQR 3230–11,680) than in G1 (2650 pg/mg; IQR 1730–8940) and in G2 (2470 pg/mg; IQR 1840–4280) (Figure 1). Differences in uDkk3/creat. were significant when G2 was compared with G3 and with G5 (*p* = 0.002 and *p* = 0.04, respectively). Table 1 shows a comparison of anthropometric and biochemical parameters across all 5 groups. The differences were assessed by the Kruskal–Wallis test and were significant for both sDkk3 and uDkk3 (*p* = 0.016 and *p* < 0.001, respectively), but not for uDkk3/creat. (*p* = 0.38).

**Figure 2:**
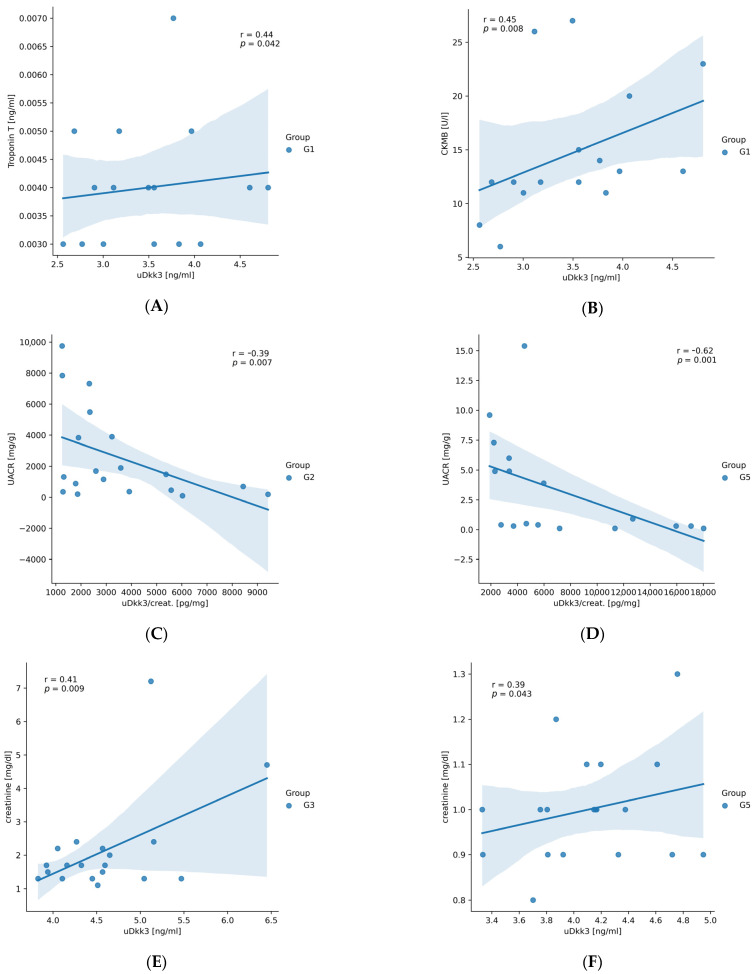
Spearman’s correlation analysis between uDkk3 and different parameters: (**A**) correlation between uDkk3 and troponin T in group 1, (**B**) correlation between uDkk3 and CK-MB in group 1, (**C**) correlation between uDkk3/creat. and UACR in group 2, (**D**) correlation between uDkk3/creat. and UACR in group 5, (**E**) correlation between uDkk3 and creatinine in group 3, (**F**) correlation between uDkk3 and creatinine in group 5. r-Spearman’s correlation coefficient; N = 20 for each group.

To verify an association between parameters, Spearman’s correlation analysis was performed. We found a correlation between uDkk3 vs. troponin T (r = 0.44, *p* = 0.042) (Figure 2A) and CK-MB (r = 0.45, *p* = 0.008) (Figure 2B) in G1, uDkk3 and CK-MB in G2 subjects (r = 0.42, *p* = 0.028). In G2 patients, the group characterized by proteinuria and normal renal function, uDkk3 and uDkk3/creat. correlated inversely with UACR (r = −0.50, *p* = 0.009 and r = −0.39, *p* = 0.007 respectively) (Figure 2C). We also found an inverse correlation between uDkk3/creat. and UACR in G5 patients (r = −0.62, *p* = 0.001) (Figure 2D). Interestingly, uDkk3 was associated with serum creatinine in G3 (r = 0.41, *p* = 0.009) (Figure 2E) and G5 patients (r = 0.39, *p* = 0.043) (Figure 2F), i.e., those with more advanced CKD. These results together with the fact that the highest uDkk3/creat. values were found in G3 and G5 suggest that urinary Dkk3 may indicate ongoing renal injury and can reflect the advancement of kidney damage. Only in G2 uDkk3/creat. correlated with age (r = −0.42, *p* = 0.004).

## 4. Discussion

Recently published data suggest that urinary Dkk3 is a sensitive indicator of ongoing tubular injury independent of eGFR and albuminuria [1,3]. Sustained increase of this protein via WNT/β-catenin pathway activation alters TEC phenotype and promotes their transformation into pro-fibrotic and pro-inflammatory phenotype. That in turn triggers EMT, leads to irreversible changes in kidney structure and results in the development and progression of CKD [1,3,5,6]. Our results demonstrate that uDkk3/creat. level is the highest in G3, a group with advanced CKD not yet on RRT (mean eGFR 32.8 ± 11.3 mL/min/1.73 m^2^). uDkk3/creat. levels in patients with eGFR ≥ 60 mL/min/1.73 m^2^ (G1, G2, and G5) were much lower, which is in agreement with the above-mentioned hypothesis and with results published previously by other research groups. In the study conducted by Zewinger et al. median uDkk3/creat. value was also higher in the CKD cohort than in the general population (431 versus 33 pg/mg). Moreover, they observed that urinary Dkk3/creat. level above 4000 pg/mg was independently associated with a mean annual decline in eGFR of 7.6% over 12 months [1]. In our study median uDkk3/creat. exceeded this threshold in G3 and G5 patients (7050 pg/mg and 5710 pg/mg, respectively). In the case of G3, it may point to an advanced damage of TEC and progressive renal fibrosis, as well as reflect poor prognosis. As G5 is a group after KTx but with otherwise preserved GFR, we hypothesize that in this case elevation of uDkk3 may reflect the ongoing albeit not yet clinically apparent renal damage and predict the long-term risk of CKD and future impaired graft function (in KTx patients TEC injury is always multifactorial and may include chronic immunological response [‘chronic rejection’], calcineurin inhibitor toxicity, arterial hypertension, and many others [16]). Of note, UACR in KTx patients was well below not only the threshold defined as microalbuminuria, but within the range defined as ‘low-grade albuminuria’ (i.e., 3 mg/g) which confirms the excellent graft health in studied transplant patients. Moreover, our study revealed a correlation between uDkk3 and serum creatinine level only in G3 and G5, which supports the potential association between uDkk3 and progressive renal function impairment in those two particular groups.

UACR is a marker of endothelial dysfunction, commonly used in everyday practice [17,18]. Schunk et al. reported that uDkk3/creat. correlated with albuminuria in patients with CKD but not in individuals from the general population. UACR > 300 mg/g was associated with higher uDKK3/creat. levels than UACR < 30 mg/g, although 48.6% of subjects with higher albuminuria had low or even undetectable levels of uDkk3 [3]. Some other studies demonstrated no correlation between uDkk3 and proteinuria in CKD cohorts [12,19]. We observed an inverse correlation between uDkk3 and UACR in G2 (the only proteinuric group) and G5 (despite the fact that UACR was extremely low in this group). Taking into account that the molecular mass of the circulating glycosylated form of Dkk3 and albumin are similar (i.e., 60–70 kDa vs. 67 kDa) [3], it seems ambiguous why urinary excretion of these proteins is so different. A likely explanation is that increased leakage through the damaged glomerular filtration barrier is further modified by tubular reabsorption, fine-tuning the final excretion. Since uDkk3 is mostly derived from tubular secretion, it may not necessarily be linked with its serum concentration. Further research is needed to determine factors that affect glomerular filtration and tubular handling of both proteins.

Piek et al. described sDkk3 as the predictor of a new-onset CKD in patients with low urinary albumin excretion (<6.6 mg/24 h) [20]. In our study, sDkk3 correlated with CK-MB and LDL in G1, i.e., a group of patients with low albuminuria (median UACR 2.7 mg/g) and without cardiovascular disease or even the risk for CVD. This correlation was not confirmed in other patient groups. As Dkk3 is also known for its proatherosclerotic properties [13], more detailed studies are needed to confirm and elucidate its significance.

Our study suffers from several limitations. One of them is a cross-sectional nature of observation with a lack of follow-up. Cardiovascular status assessment was based only on cardiac biomarkers and PWV, with only a few patients with echocardiography assessment available. The size of each subgroup (N = 100 in total, 20 subjects each) is low, nevertheless, to the best of our knowledge, this is the first study that comprehensively addressed the value of serum and urine Dkk3 as novel biomarker in patients within the whole spectrum of renal disease. It is also important that in all patients from the G2 group we established an exact (biopsy-based) diagnosis of CKD (and we did so for most of the G5 group subjects).

## 5. Conclusions

Urinary Dkk3 level was the highest in participants with advanced CKD and correlated with serum creatinine level and eGFR only in individuals with CKD and those with functioning kidney grafts. Our results confirm the potential role of Dkk3 as a biomarker of an ongoing renal injury. Future studies are needed to assess the utility of this protein in the short and long-term prognosis of CKD progression.

## Figures and Tables

**Table 1:** Comparison of anthropometric and biochemical parameters across all study groups. Data with normal distribution are presented as mean ± standard deviations, and data with non-normal distribution—as median and interquartile ranges. *p*-values were calculated using the Kruskal–Wallis test; *—*p* < 0.05, #—*p* < 0.01, $—*p* < 0.005, &—*p* < 0.001.

Parameter	Unit	G1	G2	G3	G4	G5	*p*-Value
Age	[years]	30.5 (28.0; 38.0)	56.0 (39.0; 63.0)	69.1 ± 11.0	59.3 ± 14.3	50.5 ± 12.3	<0.001 &
Weight	[kg]	71.0 ± 13.6	83.7 ± 12.5	82.5 ± 15.4	75.9 ±12.7	77.4 ± 12.8	0.026
BMI	[kg/m^2^]	24.3 ± 3.7	27.0 ± 4.0	28.7 (26.4; 31.2)	26.1 (23.9; 28.5)	26.9 ± 3.2	0.003 $
SBP	[mmHg]	122 ± 8	141 ± 20	144 ± 17	145 ± 20	133 ± 15	<0.001 &
DBP	[mmHg]	77 (70; 85)	86 ± 13	84 ± 12	81 ± 12	83 (80; 90)	0.068
HR	[beats/min]	75.4 ± 10.1	78.0 (70.0; 81.0)	75.7 ± 13.2	78.0 (76.0; 80.0)	76.8 ± 7.9	0.596
PWV	[m/s]	7.57 ± 1.27	8.54 ± 1.80	11.56 ± 1.77	8.30 (7.65; 9.45)	9.41 ± 2.13	<0.001 &
Total Cholesterol	[mg/dL]	179 ± 28	212 (184; 259)	169 ± 40	148 ± 29	171 ± 33	<0.001 &
LDL	[mg/dL]	104 ± 32	143 (112; 178)	102 ± 27	87 ± 29	84 ± 23	<0.001 &
HDL	[mg/dL]	64 ± 17	56 (46; 67)	52 ± 12	45 ± 16	65 ± 19	0.005 #
Triglycerides	[mg/dL]	101 ± 38	160 (94; 184)	138 (112; 191)	168 ± 71	139 (127; 169)	0.003 $
Creatinine	[mg/dL]	0.85 ± 0.11	0.90 (0.80; 1.00)	1.70 (1.30; 2.25)	8.22 ± 2.71	1.00 (0.90; 1.03)	<0.001 &
eGFR	[ml/min]	86.7 (79.0; 97.5)	85.7 ± 23.7	32.8 ± 11.3	6.3 (4.7; 8.3)	69.4 (62.6; 82.9)	<0.001 &
Urea	[mg/dL]	26.9 ± 6.2	36.2 ± 11.5	60.5 (54.3; 94.5)	118.1 ± 36.5	36.5 ± 7.5	<0.001 &
UACR	[mg/g]	2.7 (1.3; 2.7)	960.6 (369.3; 2238.1)	13.3 (3.5; 556.6)	-	3.5 (1.3; 5.3)	<0.001 &
Troponin T	[ng/mL]	0.0040 (0.0030; 0.0043)	0.0080 (0.0048; 0.014)	0.016 (0.009; 0.033)	0.053 (0.036; 0.083)	0.009 (0.007; 0.014)	<0.001 &
CK-MB	[U/L]	12.5 (11.0; 16.3)	14.0 (12.0; 17.0)	14.0 (12.0; 18.3)	11.58 ± 3.20	17.6 ± 5.6	0.002 $
NT-proBNP	[pg/mL]	40 ± 19	95 (38; 261)	457 (237; 2896)	6420 (3317; 14,187)	141 (107; 164)	<0.001 &
sDkk3	[ng/mL]	133 ± 117	34 (30; 39)	40 (30; 81)	50 (42; 98)	46 (36; 70)	0.016 *
uDkk3	[ng/mL]	3.33 ± 0.90	4.16 ± 0.66	4.57 (4.19; 5.10)	-	4.11 ± 0.44	<0.001 &
uDkk3/creat.	[pg/mg]	2650 (1730; 8940)	2470 (1840; 4280)	7050 (5090; 11,730)	-	5710 (3230; 11,680)	0.38

## Data Availability

Data sharing is not applicable to this article.
